# Arabidopsis glutamate receptor *GLR3.7* is involved in abscisic acid response

**DOI:** 10.1080/15592324.2021.1997513

**Published:** 2021-11-12

**Authors:** Pei-Yuan Chen, Chun-Yi Hsu, Cheng-En Lee, Ing-Feng Chang

**Affiliations:** a Institute of Plant Biology, National Taiwan University, Taipei, Taiwan; b Department of Life Science, National Taiwan University, Taipei, Taiwan; c Genome and Systems Biology Degree Program, National Taiwan University, Taipei, Taiwan

**Keywords:** Arabidopsis, abscisic acid, calcium, seed germination, glutamate receptor, salt, 14-3-3

## Abstract

The ionotropic glutamate receptor (iGluR) plays an important role in neuronal signaling in animal cells. There are at least 20 glutamate receptor-like (GLR) genes in *Arabidopsis thaliana*. These genes are involved in seed germination, root growth, wounding response, stomata closure, *etc*. A recent study showed that Arabidopsis clade III glutamate receptor *GLR3.7* is involved in salt stress response. We tested whether *GLR3.7* is involved in abscisic acid (ABA) response. In the present study, we found that the expression of *GLR3.7* was reduced by ABA treatment. Under ABA-treated condition, *GLR3.7* overexpression lines exhibited significantly higher seed germination rate at 60, 72 and 84 h under ABA-treated condition. A point mutation in 14-3-3 binding site of GLR3.7 in GLR3.7-S860A overexpression lines exhibited higher seed germination inhibition under ABA-treated conditions. Our results support that *GLR3.7* is involved in ABA response in Arabidopsis. In addition, Ser-860 of GLR3.7 appears to be important in ABA response.

In the Arabidopsis genome, there are various genes encoding ion channels including aquaporins.^1,2^ To maintain calcium concentration in the cytoplasm, the calcium transporters maintain dynamic equilibrium to calcium ions. Glutamate receptor-like channels (GLRs) are known as calcium-permeable channels.^3^ In mammals, glutamate receptors iGLuRs play essential roles in cell-to-cell communication in the nervous system.

Plant GLRs are known in regulating salt stress response. *GLR3.4* was found to be involved in seed germination under salt stress conditions^4^ in Arabidopsis. A recent study showed that Arabidopsis clade III glutamate receptor *GLR3.7* is involved in seed germination under salt stress condition.^5^ Mutant line *glr3.7–2* reduced cytosolic calcium level by salt stress. In addition, point-mutation of 14-3-3 binding site of GLR3.7 to alanine (GLR3.7-S860A) reduced root growth inhibition by salt stress.^5^

Under ABA-treated conditions, seed germination is inhibited and cotyledon greening is reduced. It was reported that *GLR1.1*-deficient mutant increased ABA sensitivity^6^ in Arabidopsis. Overexpression of *GLR3.5* reduced ABA sensitivity and seed germinated earlier in Arabidopsis.^7^ In the present study, we hypothesized and tested whether *GLR3.7* is involved in abscisic acid (ABA)-mediated seed germination in Arabidopsis. To investigate whether gene expression of *GLR3.7* is regulated by ABA, quantitative RT-PCR (qRT-PCR) was used to analyze *GLR3.7* gene expression in wild-type Col-0. The expression level of *GLR3.7* is lower under 0.5 μM and 10 μM ABA-treated condition (Figure 1a and 1b). The ABA response marker genes, *RAB18* and *RD29B*, were up-regulated under 0.5 μM and 10 μM ABA-treated condition, which indicates the ABA treatment worked (Figure 1a and 1b). These results indicated that *GLR3.7* expression is negatively regulated by the ABA in Arabidopsis.

**Figure 1.**
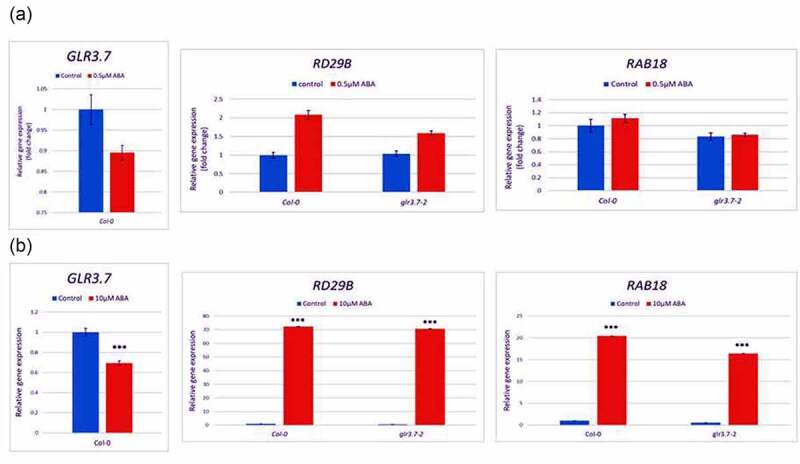
*GLR3.7* gene expression under ABA treatment. Seedlings of five-day-old wild-type were grown on ½ MS medium, and then treated with ABA or Ethanol (EtOH) for 2 hours before RNA extraction. (a) Expression level of *GLR3.7, RD29B* and *RAB18* treated with 100% EtOH (control) and 0.5 μM ABA. (b) Expression level of *GLR3.7, RD29B* and *RAB18* treated with 100% EtOH (control) and 10 μM ABA. *RAB18* and *RD29B* are ABA-related marker genes. Gene expression level of *UBQ10* was used as an internal control. These results indicated that *GLR3.7* might be negatively regulated in ABA stress response. Each bar indicates mean ± SD in three biological replicates (N≧3, n≧9). Asterisks (*) indicate significant different compared with EtOH (control) (*p < 0.05, **p < 0.01, ***p < 0.001; *Student’s t-test*).

To study if *GLR3.7* is involved in the ABA response, T-DNA insertion mutant *glr3.7–2*^5^ was introduced. In addition, *GLR3.7* overexpression lines OE5-6, OE16-5 and phosphorylation site point-mutation lines SA10-2, SA15-6^5^ (Supplemental Figure 1) were also introduced. These lines were subjected to 0.5 μM ABA treatment for seed germination assay. Our results showed that OE5-6, OE16-5 exhibited significantly higher seed germination rate at 60 h (Figs. 2, 3a), 72 h (Figs. 2, 3b) and 84 h (Figs. 2, 3c) (Supplemental table 1). By contrast, GLR3.7-S860A overexpression lines 10–2 and 15–6^5^ exhibited higher seed germination inhibition under ABA-treated conditions with significant difference from OE5-6 and OE16-5 at 72 h (Figs. 2, 3b) and 84 h (Figs. 2, 3c)(Supplemental table 1), but did not show significant difference from the wild type and *glr3.7–2*. These indicate that *GLR3.7* is involved in the ABA response in Arabidopsis.

**Figure 2.**
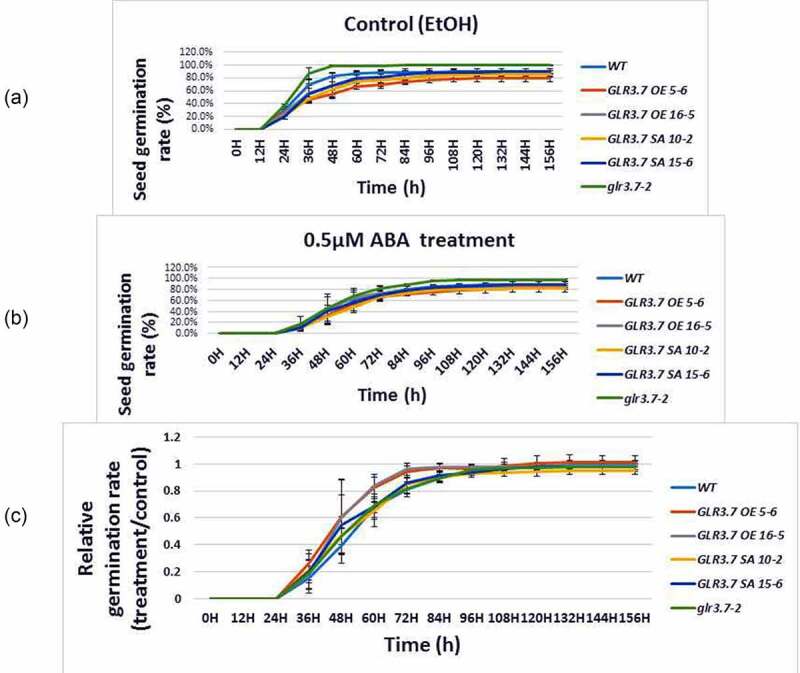
Seed germination of the *GLR3.7* overexpression lines are less sensitive to ABA than that of Col-0. Seeds of both lines were plated on half-strength MS medium containing (a) 100% EtOH (control) or (b) 0.5 μM ABA for seed germination. Germination rate was observed every 12 h. (c) Quantitative statistical analysis of seed germination rate: germination rate in the ABA-treated group divided by the germination rate in the control group. The results were statistically analyzed by Student t test (mean ± SD, N = 3, total n ≥ 600, *P < 0.05).

**Figure 3.**
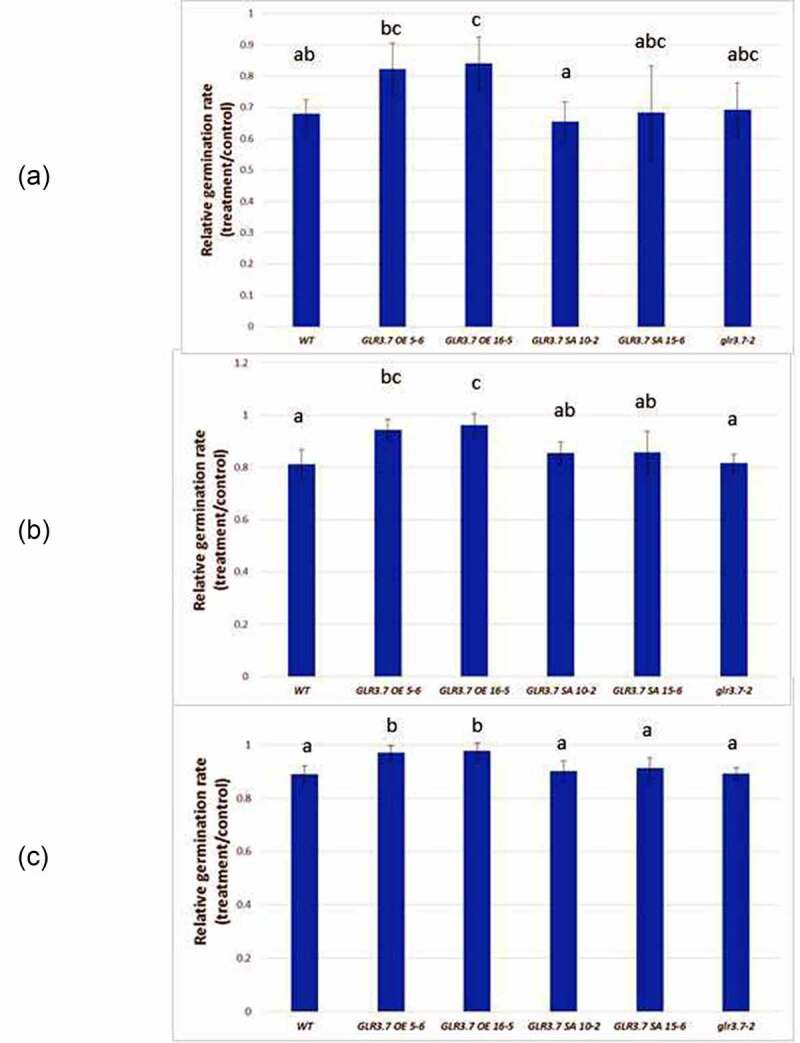
Relative seed germination rate of the *GLR3.7* overexpression lines are less sensitive to ABA than that of Col-0 and GLR3.7-S860A overexpression lines in ABA-treated (a) 60, (b) 72, (c) 84 hours. Seeds of both lines were plated on half-strength MS medium containing 100% EtOH (control) or 0.5 μM ABA for seed germination. Quantitative statistical analysis of seed germination rate: germination rate in the ABA-treated group divided by the germination rate in the control group. The results were statistically analyzed by one-way ANOVA with *post hoc* Tukey honestly significant difference test (significantly at *P* < .05). (mean ± SD, N = 3, total n ≥ 600, significantly at *P* < 0.05).

*GLR3.4* was found to be involved in seed germination under salt stress conditions^4^ in Arabidopsis. *glr3.4* mutation line exhibited higher germination sensitivity under 150 mM NaCl treatment, by delaying germination time, than the wild type.^4^ In Cheng’s study, they found that *GLR3.4* is involved in ABA-mediated seed germination.^4^ In the present study, we also found that *GLR3.7* is involved in ABA-mediated seed germination. It appeared that *GLR3.4* and *GLR3.7* can have functional redundancy. It will be interesting to cross *glr3.4* and *glr3.7* mutants to generate *glr3.4 glr3.7* double mutant and test for salt and ABA responses to confirm the functional redundancy issue. Moreover, in our previous study, we found that GLR3.7-S860A overexpression lines 10–2 and 15–6 exhibited lower sensitivity of primary root growth inhibition to salt stress.^5^ In the present study, we found that GLR3.7-S860A overexpression lines 10–2 and 15–6 exhibited higher seed germination inhibition under ABA-treated conditions, which suggests that the 14-3-3 binding site Ser-860 is important in the ABA response in Arabidopsis.

## Supplementary Material

Supplemental Material
