# Supplementary material for: Arabidopsis glutamate receptor GLR3.7 is involved in abscisic acid response
Source: Plant Signal Behav. 2021 Nov 12;16(12):1997513. doi: 10.1080/15592324.2021.1997513 (PMC9208785; doi:10.1080/15592324.2021.1997513)
Supplement: Supplemental Material [file KPSB_A_1997513_SM4477.zip › Supplemental table 1.pdf]

EtOH (control)
(Replicate 1)

	WT	GLR3.7 OE 5-6	GLR3.7 OE 16-5	GLR3.7 SA 10-2	GLR3.7 SA 15-6	*glr3.7-2*
0H	0%	0%	0%	0%	0%	0%
12H	0%	0%	0%	0%	0%	0%
24H	27%	28%	20%	16%	20%	39%
36H	66%	53%	68%	56%	68%	80%
48H	85%	60%	80%	73%	89%	98%
60H	87%	67%	85%	82%	90%	100%
72H	88%	69%	85%	83%	90%	100%
84H	89%	74%	87%	85%	93%	100%
96H	89%	76%	88%	86%	94%	100%
108H	89%	78%	88%	86%	94%	100%
120H	89%	78%	88%	86%	94%	100%
132H	89%	78%	88%	86%	94%	100%
144H	89%	78%	88%	86%	94%	100%
156H	89%	78%	88%	86%	94%	100%

EtOH (control)
(Replicate 2)

	WT	GLR3.7 OE 5-6	GLR3.7 OE 16-5	GLR3.7 SA 10-2	GLR3.7 SA 15-6	*glr3.7-2*
0H	0%	0%	0%	0%	0%	0%
12H	0%	0%	0%	0%	0%	0%
24H	28%	27%	25%	19%	23%	35%
36H	62%	41%	72%	51%	70%	79%
48H	80%	44%	84%	69%	90%	98%
60H	83%	60%	89%	77%	93%	99%
72H	85%	63%	91%	78%	94%	99%
84H	86%	69%	92%	78%	95%	99%
96H	87%	71%	93%	84%	95%	99%
108H	87%	72%	93%	87%	95%	99%
120H	87%	72%	93%	89%	95%	99%
132H	87%	72%	93%	89%	95%	99%
144H	87%	72%	93%	89%	95%	99%
156H	87%	72%	93%	89%	95%	99%

EtOH (control)
(Replicate 3)

	WT	GLR3.7 OE 5-6	GLR3.7 OE 16-5	GLR3.7 SA 10-2	GLR3.7 SA 15-6	*glr3.7-2*
0H	0%	0%	0%	0%	0%	0%
12H	0%	0%	0%	0%	0%	0%
24H	33%	31%	32%	27%	25%	37%
36H	67%	44%	67%	54%	64%	77%
48H	86%	59%	82%	74%	83%	97%
60H	91%	65%	88%	78%	86%	98%
72H	93%	70%	89%	81%	89%	99%
84H	93%	72%	92%	83%	90%	100%
96H	94%	73%	93%	84%	90%	100%
108H	94%	73%	93%	85%	90%	100%
120H	94%	73%	93%	85%	90%	100%
132H	94%	73%	93%	85%	90%	100%
144H	94%	73%	93%	85%	90%	100%
156H	94%	73%	93%	85%	90%	100%

EtOH (control)
(Replicate 4)

	WT	GLR3.7 OE 5-6	GLR3.7 OE 16-5	GLR3.7 SA 10-2	GLR3.7 SA 15-6	*glr3.7-2*
0H	0%	0%	0%	0%	0%	0%
12H	0%	0%	0%	0%	0%	0%
24H	35%	25%	22%	15%	17%	39%
36H	70%	42%	41%	39%	44%	92%
48H	78%	53%	52%	48%	51%	99%
60H	87%	71%	59%	70%	78%	100%
72H	88%	72%	60%	78%	81%	100%
84H	88%	75%	69%	82%	83%	100%
96H	88%	81%	78%	83%	87%	100%
108H	88%	83%	81%	84%	89%	100%
120H	88%	85%	82%	85%	90%	100%
132H	88%	86%	82%	85%	91%	100%
144H	88%	86%	82%	85%	92%	100%
156H	88%	86%	82%	85%	92%	100%

EtOH (control)
(Replicate 5)

	WT	GLR3.7 OE 5-6	GLR3.7 OE 16-5	GLR3.7 SA 10-2	GLR3.7 SA 15-6	*glr3.7-2*
0H	0%	0%	0%	0%	0%	0%
12H	0%	0%	0%	0%	0%	0%
24H	29%	27%	24%	19%	15%	36%
36H	80%	48%	44%	42%	40%	97%
48H	87%	57%	55%	52%	49%	100%
60H	90%	72%	65%	65%	67%	100%
72H	90%	73%	67%	72%	70%	100%
84H	90%	76%	70%	78%	76%	100%
96H	90%	82%	77%	82%	80%	100%
108H	90%	85%	78%	84%	82%	100%
120H	90%	87%	80%	85%	83%	100%
132H	90%	87%	80%	85%	86%	100%
144H	90%	87%	80%	85%	86%	100%
156H	90%	87%	80%	85%	86%	100%

EtOH (control)
(Replicate 6)

	WT	GLR3.7 OE 5-6	GLR3.7 OE 16-5	GLR3.7 SA 10-2	GLR3.7 SA 15-6	*glr3.7-2*
0H	0%	0%	0%	0%	0%	0%
12H	0%	0%	0%	0%	0%	0%
24H	33%	29%	33%	29%	21%	37%
36H	77%	46%	49%	48%	43%	95%
48H	84%	57%	58%	58%	50%	99%
60H	88%	69%	66%	69%	65%	99%
72H	88%	71%	67%	71%	68%	99%
84H	88%	74%	72%	77%	77%	99%
96H	88%	80%	80%	81%	83%	99%
108H	89%	83%	82%	81%	83%	100%
120H	89%	84%	84%	82%	84%	100%
132H	89%	84%	84%	82%	85%	100%
144H	89%	85%	84%	82%	85%	100%
156H	89%	85%	84%	82%	85%	100%

0.5µM ABA
(Replicate 1)

	WT	GLR3.7 OE 5-6	GLR3.7 OE 16-5	GLR3.7 SA 10-2	GLR3.7 SA 15-6	*glr3.7-2*
0H	0%	0%	0%	0%	0%	0%
12H	0%	0%	0%	0%	0%	0%
24H	0%	0%	0%	0%	0%	0%
36H	3%	8%	5%	7%	7%	4%
48H	41%	42%	68%	40%	70%	48%
60H	52%	55%	78%	56%	75%	58%
72H	73%	62%	87%	66%	83%	82%
84H	82%	70%	88%	73%	88%	90%
96H	87%	72%	88%	77%	90%	95%
108H	89%	73%	88%	77%	92%	98%
120H	89%	74%	89%	77%	92%	99%
132H	89%	75%	89%	78%	93%	99%
144H	89%	75%	89%	78%	93%	99%
156H	89%	75%	89%	78%	93%	99%

0.5µM ABA
(Replicate 2)

	WT	GLR3.7 OE 5-6	GLR3.7 OE 16-5	GLR3.7 SA 10-2	GLR3.7 SA 15-6	*glr3.7-2*
0H	0%	0%	0%	0%	0%	0%
12H	0%	0%	0%	0%	0%	0%
24H	0%	0%	0%	0%	0%	0%
36H	7%	14%	6%	8%	11%	6%
48H	41%	44%	69%	41%	58%	51%
60H	56%	57%	82%	51%	71%	63%
72H	76%	60%	87%	70%	79%	81%
84H	80%	65%	89%	75%	82%	91%
96H	85%	67%	89%	80%	86%	95%
108H	86%	69%	90%	81%	90%	95%
120H	88%	72%	91%	82%	90%	95%
132H	89%	74%	91%	83%	91%	95%
144H	89%	74%	91%	83%	91%	95%
156H	89%	74%	91%	83%	91%	95%

0.5µM ABA
(Replicate 3)

	WT	GLR3.7 OE 5-6	GLR3.7 OE 16-5	GLR3.7 SA 10-2	GLR3.7 SA 15-6	*glr3.7-2*
0H	0%	0%	0%	0%	0%	0%
12H	0%	0%	0%	0%	0%	0%
24H	0%	0%	0%	0%	0%	0%
36H	7%	10%	9%	12%	13%	3%
48H	44%	48%	72%	46%	65%	53%
60H	62%	58%	79%	54%	72%	62%
72H	78%	71%	85%	70%	81%	77%
84H	81%	72%	87%	72%	84%	87%
96H	84%	73%	89%	76%	85%	94%
108H	89%	78%	89%	78%	88%	96%
120H	90%	80%	90%	81%	91%	96%
132H	90%	80%	91%	82%	92%	96%
144H	90%	80%	91%	82%	92%	96%
156H	90%	80%	91%	82%	92%	96%

0.5µM ABA
(Replicate 4)

	WT	GLR3.7 OE 5-6	GLR3.7 OE 16-5	GLR3.7 SA 10-2	GLR3.7 SA 15-6	*glr3.7-2*
0H	0%	0%	0%	0%	0%	0%
12H	0%	0%	0%	0%	0%	0%
24H	0%	0%	0%	0%	0%	0%
36H	20%	9%	9%	13%	8%	33%
48H	28%	16%	16%	21%	14%	45%
60H	60%	54%	48%	43%	37%	78%
72H	71%	68%	60%	63%	57%	79%
84H	79%	75%	70%	73%	72%	86%
96H	84%	80%	78%	79%	81%	94%
108H	87%	85%	80%	81%	83%	96%
120H	90%	87%	80%	83%	85%	97%
132H	91%	88%	80%	83%	86%	98%
144H	91%	88%	80%	83%	87%	98%
156H	91%	88%	80%	83%	87%	98%

0.5μM ABA
(Replicate 5)

	WT	GLR3.7 OE 5-6	GLR3.7 OE 16-5	GLR3.7 SA 10-2	GLR3.7 SA 15-6	*glr3.7-2*
0H	0%	0%	0%	0%	0%	0%
12H	0%	0%	0%	0%	0%	0%
24H	0%	0%	0%	0%	0%	0%
36H	18%	12%	10%	11%	14%	27%
48H	23%	20%	17%	18%	22%	40%
60H	65%	54%	50%	47%	40%	76%
72H	68%	68%	61%	64%	62%	83%
84H	78%	73%	68%	71%	70%	90%
96H	84%	76%	77%	76%	75%	98%
108H	84%	81%	80%	79%	79%	99%
120H	85%	83%	81%	80%	81%	99%
132H	86%	83%	81%	80%	81%	100%
144H	86%	83%	81%	80%	81%	100%
156H	86%	83%	81%	80%	81%	100%

0.5μM ABA
(Replicate 6)

	WT	GLR3.7 OE 5-6	GLR3.7 OE 16-5	GLR3.7 SA 10-2	GLR3.7 SA 15-6	*glr3.7-2*
0H	0%	0%	0%	0%	0%	0%
12H	0%	0%	0%	0%	0%	0%
24H	0%	0%	0%	0%	0%	0%
36H	12%	19%	17%	9%	9%	29%
48H	19%	29%	27%	17%	17%	39%
60H	63%	53%	48%	38%	39%	76%
72H	66%	65%	62%	62%	60%	86%
84H	76%	73%	69%	71%	73%	90%
96H	86%	78%	76%	78%	79%	95%
108H	87%	82%	78%	80%	82%	96%
120H	87%	86%	79%	81%	84%	97%
132H	87%	86%	79%	81%	87%	97%
144H	87%	86%	79%	81%	87%	97%
156H	87%	86%	79%	81%	87%	97%

Normalized
(Replicate 1)

	WT	GLR3.7 OE 5-6	GLR3.7 OE 16-5	GLR3.7 SA 10-2	GLR3.7 SA 15-6	*glr3.7-2*
0H	0	0	0	0	0	0
12H	0	0	0	0	0	0
24H	0	0	0	0	0	0
36H	4.55%	0.150943396	0.073529412	0.125	0.102941176	0.05
48H	0.48235	0.7	0.85	0.547945205	0.786516854	0.4898
60H	0.5977	0.820895522	0.917647059	0.682926829	0.833333333	0.58
72H	0.82955	0.898550725	1.023529412	0.795180723	0.922222222	0.82
84H	0.92135	0.945945946	1.011494253	0.858823529	0.946236559	0.9
96H	0.97753	0.947368421	1	0.895348837	0.957446809	0.95
108H	1	0.935897436	1	0.895348837	0.978723404	0.98
120H	1	0.948717949	1.011363636	0.895348837	0.978723404	0.99
132H	1	0.961538462	1.011363636	0.906976744	0.989361702	0.99
144H	1	0.961538462	1.011363636	0.906976744	0.989361702	0.99
156H	1	0.961538462	1.011363636	0.906976744	0.989361702	0.99

Normalized
(Replicate 2)

	WT	GLR3.7 OE 5-6	GLR3.7 OE 16-5	GLR3.7 SA 10-2	GLR3.7 SA 15-6	*glr3.7-2*
0H	0	0	0	0	0	0
12H	0	0	0	0	0	0
24H	0	0	0	0	0	0
36H	0.1129	0.341463415	0.083333333	0.156862745	0.157142857	0.07595
48H	0.5125	1	0.821428571	0.594202899	0.644444444	0.52041
60H	0.6747	0.95	0.921348315	0.662337662	0.76344086	0.63636
72H	0.89412	0.952380952	0.956043956	0.897435897	0.840425532	0.81818
84H	0.93023	0.942028986	0.967391304	0.961538462	0.863157895	0.91919
96H	0.97701	0.943661972	0.956989247	0.952380952	0.905263158	0.9596
108H	0.98851	0.958333333	0.967741935	0.931034483	0.947368421	0.9596
120H	1.01149	1	0.978494624	0.921348315	0.947368421	0.9596
132H	1.02299	1.027777778	0.978494624	0.93258427	0.957894737	0.9596
144H	1.02299	1.027777778	0.978494624	0.93258427	0.957894737	0.9596
156H	1.02299	1.027777778	0.978494624	0.93258427	0.957894737	0.9596

Normalized
(Replicate 3)

	WT	GLR3.7 OE 5-6	GLR3.7 OE 16-5	GLR3.7 SA 10-2	GLR3.7 SA 15-6	*glr3.7-2*
0H	0	0	0	0	0	0
12H	0	0	0	0	0	0
24H	0	0	0	0	0	0
36H	0.10448	0.227272727	0.134328358	0.222222222	0.203125	0.03896
48H	0.51163	0.813559322	0.87804878	0.621621622	0.78313253	0.54639
60H	0.68132	0.892307692	0.897727273	0.692307692	0.837209302	0.63265
72H	0.83871	1.014285714	0.95505618	0.864197531	0.91011236	0.77778
84H	0.87097	1	0.945652174	0.86746988	0.933333333	0.87
96H	0.89362	1	0.956989247	0.904761905	0.944444444	0.94
108H	0.94681	1.068493151	0.956989247	0.917647059	0.977777778	0.96
120H	0.95745	1.095890411	0.967741935	0.952941176	1.011111111	0.96
132H	0.95745	1.095890411	0.978494624	0.964705882	1.022222222	0.96
144H	0.95745	1.095890411	0.978494624	0.964705882	1.022222222	0.96
156H	0.95745	1.095890411	0.978494624	0.964705882	1.022222222	0.96

Normalized
(Replicate 4)

	WT	GLR3.7 OE 5-6	GLR3.7 OE 16-5	GLR3.7 SA 10-2	GLR3.7 SA 15-6	*glr3.7-2*
0H	0	0	0	0	0	0
12H	0	0	0	0	0	0
24H	0	0	0	0	0	0
36H	28.57%	0.214285714	0.219512195	0.333333333	0.181818182	0.3587
48H	0.35897	0.301886792	0.307692308	0.4375	0.274509804	0.45455
60H	0.68966	0.76056338	0.813559322	0.614285714	0.474358974	0.78
72H	0.80682	0.944444444	1	0.807692308	0.703703704	0.79
84H	0.89773	1	1.014492754	0.890243902	0.86746988	0.86
96H	0.95455	0.987654321	1	0.951807229	0.931034483	0.94
108H	0.98864	1.024096386	0.987654321	0.964285714	0.93258427	0.96
120H	1.02273	1.023529412	0.975609756	0.976470588	0.944444444	0.97
132H	1.03409	1.023255814	0.975609756	0.976470588	0.945054945	0.98
144H	1.03409	1.023255814	0.975609756	0.976470588	0.945652174	0.98
156H	1.03409	1.023255814	0.975609756	0.976470588	0.945652174	0.98

Normalized
(Replicate 5)

	WT	GLR3.7 OE 5-6	GLR3.7 OE 16-5	GLR3.7 SA 10-2	GLR3.7 SA 15-6	*glr3.7-2*
0H	0	0	0	0	0	0
12H	0	0	0	0	0	0
24H	0	0	0	0	0	0
36H	0.225	0.25	0.227272727	0.261904762	0.35	0.27835
48H	0.26437	0.350877193	0.309090909	0.346153846	0.448979592	0.4
60H	0.72222	0.75	0.769230769	0.723076923	0.597014925	0.76
72H	0.75556	0.931506849	0.910447761	0.888888889	0.885714286	0.83
84H	0.86667	0.960526316	0.971428571	0.91025641	0.921052632	0.9
96H	0.93333	0.926829268	1	0.926829268	0.9375	0.98
108H	0.93333	0.952941176	1.025641026	0.94047619	0.963414634	0.99
120H	0.94444	0.954022989	1.0125	0.941176471	0.975903614	0.99
132H	0.95556	0.954022989	1.0125	0.941176471	0.941860465	1
144H	0.95556	0.954022989	1.0125	0.941176471	0.941860465	1
156H	0.95556	0.954022989	1.0125	0.941176471	0.941860465	1

Normalized
(Replicate 6)

	WT	GLR3.7 OE 5-6	GLR3.7 OE 16-5	GLR3.7 SA 10-2	GLR3.7 SA 15-6	*glr3.7-2*
0H	0	0	0	0	0	0
12H	0	0	0	0	0	0
24H	0	0	0	0	0	0
36H	0.15584	0.413043478	0.346938776	0.1875	0.209302326	0.30526
48H	0.22619	0.50877193	0.465517241	0.293103448	0.34	0.39394
60H	0.71591	0.768115942	0.727272727	0.550724638	0.6	0.76768
72H	0.75	0.915492958	0.925373134	0.873239437	0.882352941	0.86869
84H	0.86364	0.986486486	0.958333333	0.922077922	0.948051948	0.90909
96H	0.97727	0.975	0.95	0.962962963	0.951807229	0.9596
108H	0.97753	0.987951807	0.951219512	0.987654321	0.987951807	0.96
120H	0.97753	1.023809524	0.94047619	0.987804878	1	0.97
132H	0.97753	1.023809524	0.94047619	0.987804878	1.023529412	0.97
144H	0.97753	1.011764706	0.94047619	0.987804878	1.023529412	0.97
156H	0.97753	1.011764706	0.94047619	0.987804878	1.023529412	0.97

	WT	GLR3.7 OE 5-6	GLR3.7 OE 16-5	GLR3.7 SA 10-2	GLR3.7 SA 15-6	*glr3.7-2*
**60H**	0.5977	0.820895522	0.917647059	0.682926829	0.833333333	0.58
	0.6747	0.95	0.921348315	0.662337662	0.76344086	0.63636
	0.68132	0.892307692	0.897727273	0.692307692	0.837209302	0.63265
	0.68966	0.76056338	0.813559322	0.614285714	0.474358974	0.78
	0.72222	0.75	0.769230769	0.723076923	0.597014925	0.76
	0.71591	0.768115942	0.727272727	0.550724638	0.6	0.76768

	WT	GLR3.7 OE 5-6	GLR3.7 OE 16-5	GLR3.7 SA 10-2	GLR3.7 SA 15-6	*glr3.7-2*
**72H**	0.82955	0.898550725	1.023529412	0.795180723	0.922222222	0.82
	0.89412	0.952380952	0.956043956	0.897435897	0.840425532	0.81818
	0.83871	1.014285714	0.95505618	0.864197531	0.91011236	0.77778
	0.80682	0.944444444	1	0.807692308	0.703703704	0.79
	0.75556	0.931506849	0.910447761	0.888888889	0.885714286	0.83
	0.75	0.915492958	0.925373134	0.873239437	0.882352941	0.86869

	WT	GLR3.7 OE 5-6	GLR3.7 OE 16-5	GLR3.7 SA 10-2	GLR3.7 SA 15-6	*cdpk16 1-1*
**84H**	0.92135	0.945945946	1.011494253	0.858823529	0.946236559	0.9
	0.93023	0.942028986	0.967391304	0.961538462	0.863157895	0.91919
	0.87097	1	0.945652174	0.86746988	0.933333333	0.87
	0.89773	1	1.014492754	0.890243902	0.86746988	0.86
	0.86667	0.960526316	0.971428571	0.91025641	0.921052632	0.9
	0.86364	0.986486486	0.958333333	0.922077922	0.948051948	0.90909
