# Supplementary figures and images for: Arabidopsis glutamate receptor GLR3.7 is involved in abscisic acid response
Source: Plant Signal Behav. 2021 Nov 12;16(12):1997513. doi: 10.1080/15592324.2021.1997513 (PMC9208785; doi:10.1080/15592324.2021.1997513)

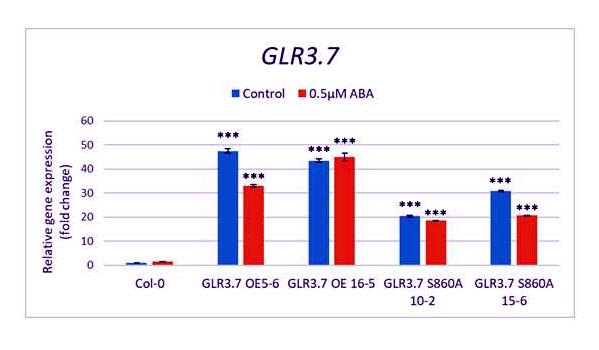

Supplement: Supplemental Material [file KPSB_A_1997513_SM4477.zip › PSB-2021-Supplemental figure 1.jpg]
